# Lenalidomide induces apoptosis and alters gene expression in non-small cell lung cancer cells

**DOI:** 10.3892/ol.2012.1054

**Published:** 2012-11-30

**Authors:** KARAM KIM, SUNGKWAN AN, HWA JUN CHA, YEONG MIN CHOI, SUNG JIN CHOI, IN-SOOK AN, HONG GHI LEE, YOO HONG MIN, SU-JAE LEE, SEUNGHEE BAE

**Affiliations:** 1 Molecular-Targeted Drug Research Center, Konkuk University, Gwangjin-gu, Seoul 143-701;; 2 Korea Institute for Skin and Clinical Sciences, Konkuk University, Gwangjin-gu, Seoul 143-701;; 3 Department of Internal Medicine, Konkuk University College of Medicine, Seoul 143-729;; 4 Department of Internal Medicine and Center for Chronic Metabolic Disease, Yonsei University College of Medicine, Seoul 102-752;; 5 Department of Chemistry, Hanyang University, Seongdong-gu, Seoul 133-791, Republic of Korea

**Keywords:** lenalidomide, non-small cell lung carcinoma cell, cell growth inhibition, gene expression profiles

## Abstract

Non-small cell lung cancer (NSCLC) is the most deadly type of cancer worldwide. Although a number of therapies are used in NSCLC treatment, their therapeutic efficacy remains low. Lenalidomide was originally approved for use in patients with myelodysplastic syndromes, which are associated with 5q deletions, and multiple myeloma. Recently, lenalidomide was investigated as a new NSCLC treatment, and it exerted anticancer effects. However, the primary cellular mechanism of its effects in NSCLC is largely unknown. Therefore, we attempted to elucidate a molecular portrait of lenalidomide-mediated cellular events in NSCLC. Lenalidomide reduced the viability of several NSCLC cell lines in a concentration-dependent manner. In addition, array-based gene expression analysis revealed that lenalidomide regulated the expression of several genes associated with cell survival, apoptosis and development, including BH3-interacting domain death agonist (BID), v-fos FBJ murine osteosarcoma viral oncogene homolog (FOS) and NK2 homeobox1 (NKX2-1). BID and FOS, which are known apoptosis activators, were upregulated by lenalidomide treatment, whereas NKX2-1, which is used as an immunohistochemistry marker for NSCLC, was downregulated. These results provide evidence that lenalidomide directly induces antiproliferative effects by altering the expression of genes associated with cell proliferation and apoptosis.

## Introduction

Lung cancer is one of the most common types of cancer and it remains the leading cause of cancer mortality in males and females worldwide. There are two major types of lung cancer: small cell lung cancer and non-small cell lung cancer (NSCLC) (1,2). NSCLC, which is the most common type of lung cancer, has a low 5-year survival rate (<15%), a poor prognosis and a high rate of relapse (3). At present, various chemotherapies, including combination therapies and targeted therapies, have been utilized to treat NSCLC; however, the efficacy of these therapies is insufficient. Therefore, the development of other therapies against NSCLC progression is required for improving the efficacy of cancer treatment.

As a novel therapeutic strategy, immunomodulatory drugs have been utilized to treat NSCLC. The immunomodulatory drug lenalidomide was originally approved to treat myelodysplastic syndromes and multiple myeloma (4,5). However, lenalidomide has been investigated for treating other types of cancer, including NSCLC, malignant melanoma and prostate cancer (6–11). Lenalidomide has been reported to alter the production of cytokines and growth factors, which in turn enhances the immune response against tumor cells and inhibits tumor angiogenesis (12,13). Moreover, it has been reported that lenalidomide is a potent co-stimulator of T-cell activation, leading to increased T-cell cytokine production and activation of CD8^+^ T cells and NK cells (14,15). Furthermore, lenalidomide has direct antiproliferative effects on tumor cells in the absence of immune effector cells (16). Lenalidomide causes concentration-dependent cell cycle arrest in G0-G1 phase by upregulating the CDK inhibitor p21 waf-1, a key cell cycle regulator that modulates the activity of CDKs, and down-regulating the activities of the prosurvival kinases ERK1/2 and Akt (17,18). Lenalidomide inhibits the translation of C/EBPβ by downregulating eIF4E, and IRF4 downregulation has been reported to be a critical factor controlling multiple myeloma survival and as a prognostic marker in patients with multiple myeloma associated with poor survival (19,20). In NSCLC, objective responses have been observed with lenalidomide-based therapy, suggesting that lenalidomide is a potent drug for NSCLC treatment. Despite the clinical effects of lenalidomide, studies of the precise mechanisms of its action in NSCLC have not yet been performed. Studies of the mechanisms of the anti-cancer properties of lenalidomide are critical for improving the efficacy of this drug against NSCLC. In the present study, we investigated the antiproliferative activity of lenalidomide against NSCLC cell lines and the gene expression profile changes in lenalidomide-treated NSCLC cells.

## Materials and methods

### Cell culture and lenalidomide treatment

The human NSCLC cell lines Lu-99, H1299, A549, EBC1 and H460 were purchased from the ATCC (Manassas, VA, USA) and cultured in RPMI-1640 medium containing 10% fetal bovine serum and antibiotics at 37°C in a humidified chamber containing 5% CO_2_. Cells were seeded into 60-mm culture dishes (2x10^5^ cells per dish) with various concentrations of lenalidomide (Celgene, Summit, NJ, USA) and incubated for various times.

### RNA preparation and cDNA synthesis

Following lenalidomide treatment, total RNA was extracted from cells using TRIzol reagent (Invitrogen, Carlsbad, CA, USA) according to the manufacturer’s instructions. For the microarray studies, both the quality and concentration of the RNA samples were determined using an Agilent 2100 Bioanalyzer (Agilent Technologies, Santa Clara, CA, USA) and a MaestroNano Spectrophotometer (Maestrogen, Las Vegas, NV, USA). The recommended RNA quality parameters for microarray analysis are as follows: UV spectroscopy A260/A280 ratio of 1.8–2.0 and an A260/A230 ratio >1.8; an 18S/28S rRNA ratio of 1.8–2.1; and an RNA integrity number >8.0. To synthesize cDNA, 1 *μ*g RNA was incubated with oligo dT primers at 94°C for 10 min and reverse transcribed with reverse transcriptase (Enzynomics, Seoul, Korea) at 37°C for 1 h.

### DNA microarray analysis

DNA microarray analysis was performed using a HumanHT-12 v4.0 Expression Beadchip kit (Illumina, San Diego, CA, USA) according to the manufacturer’s instructions. The derived data were analyzed using Genespring GX 11 (Agilent Technologies). The raw data were filtered using the flag test and t-test, therefore the miRNAs showing detectable expression levels (flag value = present) were selected, and subjected to fold-change analysis. Significant genes were determined using the fluorescence ratio between the control and lenalidomide-treated samples, and genes displaying a >2-fold increase or decrease were selected for analysis.

### Polymerase chain reaction (PCR) analysis

The expression levels of BH3-interacting domain death agonist (BID), v-fos FBJ murine osteosarcoma viral oncogene homolog (FOS) and NK2 homeobox1 (NKX2-1) mRNA were determined by PCR with their specific primers as follows: GADPH, (F) 5′-TTGCCATCAATGACCCCTTCA-3′ and (R) 5′-CGC CCCACTTGATTTTGGA-3′; BID, (F) 5′-ATGGAC TGTGAGGTCAACAACGG-3′ and (R) 5′-CACGTA GGTGCGTAGGTTCTGGTTA-3′; Fos, (F) 5′-CCAACT TCAT TCCCACG GTCAC-3′ and (R) 5′-TG GCA A TCTCGGTCTGCAAA-3′; NKX2-1, (F) 5′-ATGTCG ATGAGTCCAAAGCA-3′ and (R) 5′-ACCG TATAGCA AGGTGGAGCA-3′.

### Cell viability assay

Cell proliferation was determined using the WST-1 assay (EZ-Cytox Cell Viability Assay kit, ITSBIO, Seoul, Korea) according to the manufacturer’s instructions. In brief, cells were incubated in 96-well plates and treated with DMSO or various concentrations of lenalidomide in RPMI-1640 medium containing 10% FBS for 2 or 3 days. After incubation, the kit solution was added to the cultured cells, which were incubated at 37°C for 2 h. Cell viability was measured using an iMark microplate reader (Bio-Rad, Hercules, CA, USA) at 450 nm using a 620-nm reference filter.

### Statistical analysis

Statistical analysis was performed using the χ^2^ test or Fisher’s exact test and Spearman rank correlation coefficient analysis. P<0.05 was considered to indicate a statistically significant result.

## Results and Discussion

**Figure 1.**
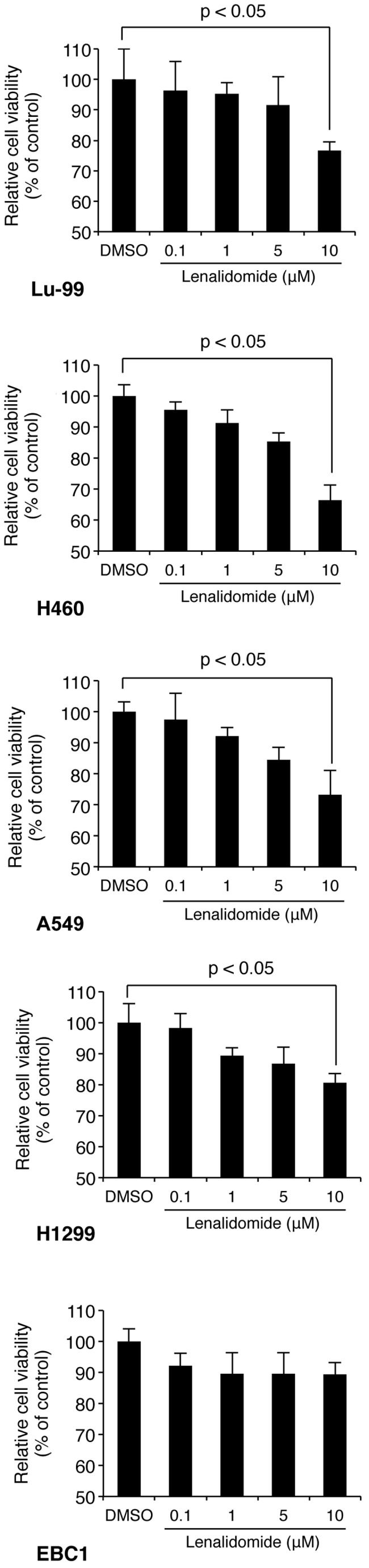
Lenalidomide induces the loss of cell viability in NSCLC cell lines. Lu-99, H1299, H460, EBC1 and A549 cells were treated with a series of concentrations of lenalidomide for 72 h. The cell viabilities of these cell lines were detected by the WST-1 assay. All experiments were performed in triplicate (n=3). Statistical significance compared with the findings in control cells for each condition was indicated by P<0.05. NSCLC, non-small cell lung cancer.

We first examined whether the immunomodulatory drug lenalidomide exhibited direct antitumor activity in NSCLC cell lines. To this end, Lu-99, H1299, H460, EBC1 and A549 cells were exposed to a series of increasing concentrations of lenalidomide for 72 h and the proliferation of the cells was measured by analyzing the activity of mitochondrial dehydrogenases using the WST-1 assay (described in Materials and methods). The assays revealed that lenalidomide significantly inhibited the proliferation of NSCLC cells (Lu-99, H1299, H460 and A549) in a concentration-dependent manner (Fig. 1). In particular, H460 cells had the highest sensitivity for lenalidomide, and therefore, we used this cell line to perform the subsequent experiments.

**Figure 2.**
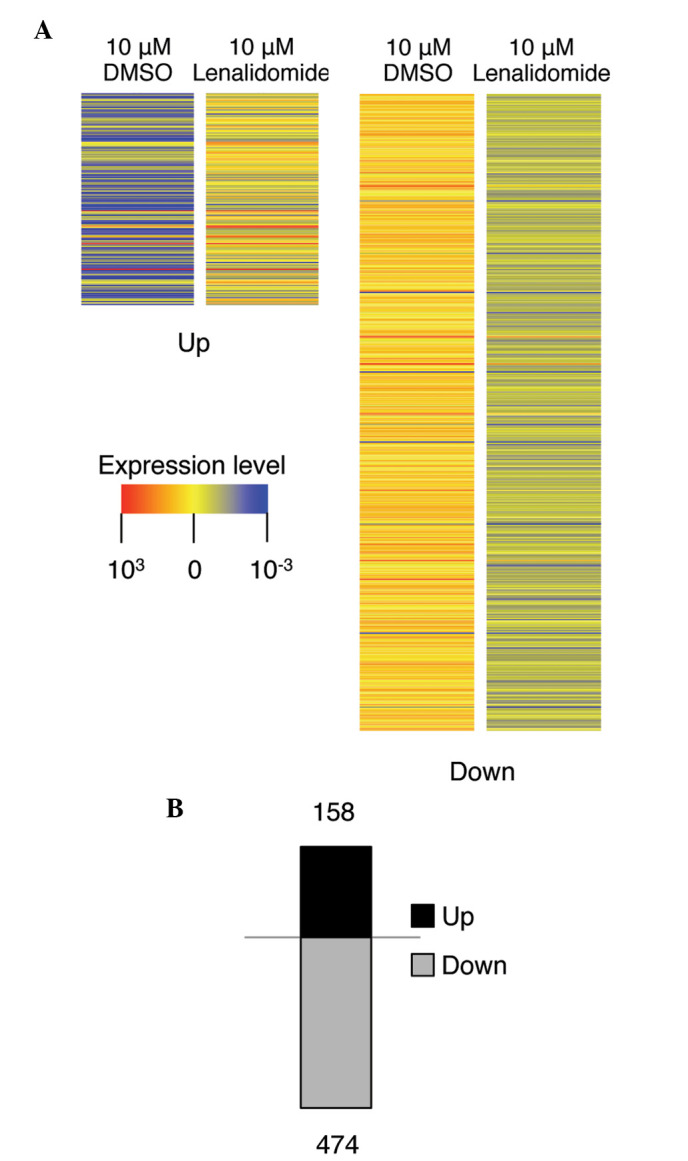
Lenalidomide causes changes in the gene expression profiles of NSCLC cells. (A) Genes that displayed >2-fold changes in expression following lenalidomide treatment compared with their expression in control cells. Genes upregulated by lenalidomide are presented on the left and genes downregulated by lenalidomide are presented on the right. (B) The numbers of genes up- and downregulated by lenalidomide treatment. NSCLC, non-small cell lung cancer.

We next performed gene expression profiling analysis to gain insight into the mode of action of lenalidomide and to identify the molecular targets of this drug. H460 cells were treated with the control DMSO or 10 *μ*M lenalidomide for 72 h and RNA samples were purified. The quality of each sample was confirmed using Agilent software. We then compared the gene expression profiles of the lenalidomide- and DMSO-treated samples (see Materials and methods). As shown in Fig. 2A, lenalidomide regulated the transcription of genes, and the genes displaying >1.5-fold changes in transcription compared with that in control cells were selected and identified by color contrast. Notably, 3-fold more mRNAs were downregulated (474 mRNAs) than upregulated (158 mRNAs) by lenalidomide treatment in H460 cells, indicating that lenalidomide has an inhibitory effect on gene transcription. To further analyze the transcriptional effect of lenalidomide, the 50 genes most strongly affected by lenalidomide treatment were selected, as shown in Table I. Furthermore, using the bioinformatics tool of Gene Set Enrichment Analysis (http://www.broadinstitute.org/gsea/index.jsp), the genes with >1.5-fold changes in expression were categorized into different functional categories, such as homology or biochemical activity, using the Gene Ontology project for gene sharing (Table II). Specifically, transcription factors, tumor suppressors and oncogenes were relatively more abundant than other biological function-related genes in Table II.

**Figure 3.**
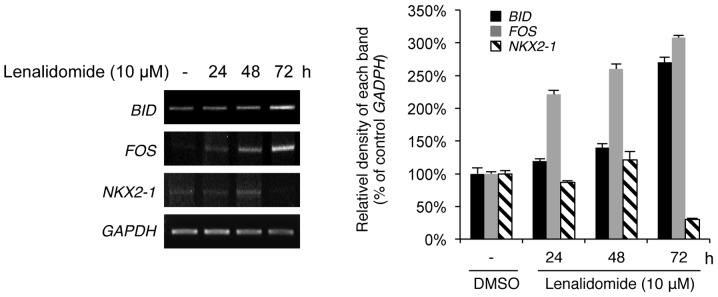
Lenalidomide regulates BID, FOS and NKX2-1 expression. A549 cells were treated with 10 *μ*M lenalidomide for 24, 48 and 72 h. After treatment, the levels of BID, FOS and NKX2-1 were determined by RT-PCR (right). The graph presents the mean ± SD of the relative intensities of BID, FOS and NKX2-1 from triplicate experiments. BID, BH3-interacting domain death agonist; FOS, v-fos FBJ murine osteosarcoma viral oncogene homolog; NKX2-1, NK2 homeobox 1.

To verify the microarray data, we performed reverse-transcription PCR (RT-PCR) analysis of several genes selected on the basis of their roles in apoptosis, cell cycle regulation, transcription and oncogenesis in NSCLC cells. These genes were BID, FOS and NKX2-1. BID is a member of the proapoptotic BCL2 family and is a key component of death receptor-mediated caspase activation (21). FOS is a transcription factor that binds to Jun and creates the transcription factor activator protein 1, which regulates cell proliferation, differentiation and apoptosis (22). Although the experimental condition of the microarray was based on the single time point (72 h) of lenalidomide treatment, as shown in Fig. 1, RT-PCR was performed to clarify the results of the expression levels of BID, FOS and NKX2-1 by treating cells with lenalidomide for 24, 48 and 72 h. As shown in Fig. 3, the results revealed good concordance in gene expression between the microarray and RT-PCR data. BID and FOS, which were upregulated by lenalidomide in the array data, were markedly upregulated in a time-dependent manner; conversely, NKX2-1 was downregulated by lenalidomide in the array and RT-PCR data. Thus, lenalidomide-induced antiproliferative effects may be mediated by regulating the expression of genes associated with apoptosis, cell survival and transcription factors.

In NSCLC, the NKX2-1 (also known as TTF1) gene is an immunohistochemical marker for predicting the adenocarcinoma subtype (23,24). The NKX2-1 gene is amplified in 10–15% of lung adenocarcinomas, and results of *in vitro* studies further support the hypothesis that NKX2-1 acts as a lineage-specific oncogene (24,25). However, a tumor suppressor role has been observed in the same type of cancer for NKX2-1 (26). The expression of exogenous NKX2-1 limited tumor progression, resulting in fewer tumors with an advanced histopathological grade. Therefore, NKX2-1 has both oncogenic and tumor-suppressive functions in lung cancer, suggesting that NKX2-1 downregulation following lenalidomide treatment in NSCLC cells is a meaningful result that requires further investigation.

These findings further the knowledge of the signaling pathways targeted by lenalidomide and suggest that lenalidomide causes changes in the gene expression profile of NSCLC cells. In addition, these genes may be prospective target molecules for the mechanism involved in the lenalidomide-induced anti-proliferative effect in NSCLC cells.

## Figures and Tables

**Table I.** Top 50 genes displaying >1.5-fold changes in expression following lenalidomide treatment.

Upregulated genes		Downregulated genes	
Gene name	FC	Gene name	FC
C14ORF153	2.92	ZNF121	2.91
GINS4	2.53	NMI	2.01
XRCC2	2.08	OR5T1	1.98
SNN	2.01	ALOX15	1.87
GRIPAP1	1.93	KCNA4	1.84
FKBP14	1.92	ARR3	1.78
BPNT1	1.87	OR11A1	1.78
RBBP9	1.85	HFM1	1.75
BLZF1	1.85	NKX2-1	1.75
HMG1L1	1.81	LOXL1	1.74
QRFPR	1.81	WDR21C	1.74
LMOD3	1.78	PENK	1.73
ZNF483	1.77	CCDC121	1.73
DCDC1	1.77	NOS2A	1.71
SHROOM4	1.77	LONRF3	1.70
DUSP19	1.76	SS18	1.70
ZNF549	1.75	AIM1L	1.69
CHRNAS	1.75	EFCBP1	1.69
DEM1	1.75	KLC3	1.68
USP49	1.74	CSAG3B	1.68
PDP2	1.74	NUCKS1	1.67
ZMAT3	1.74	MORF4L1	1.67
MYO3B	1.74	LY6G5C	1.67
ZNF69	1.73	ESRRG	1.66
OVOS2	1.73	LYPD4	1.66
LRRFIP1	1.72	LRRN3	1.66
TTTY22	1.72	KIF5A	1.66
TRIM13	1.72	FBXO47	1.66
GNB4	1.72	PRDM13	1.65
CCBE1	1.72	SYT15	1.65
ZNF14	1.71	UPK3B	1.65
FOS	1.71	CPM	1.65
IL17RD	1.70	BZW1	1.65
MAGT1	1.70	ASB4	1.64
TDP1	1.67	MEST	1.64
EIB2B	1.67	LGR6	1.63
XCL1	1.67	GEM	1.63
CDKN2A	1.67	MICALL2	1.62
BID	1.66	PCDHGA1	1.62
DDX51	1.65	PCLO	1.62
ARL16	1.63	SYPL2	1.62
AGER	1.63	NR1I2	1.61
NLRP8	1.63	EPHA10	1.61
FUT6	1.61	DGKB	1.61
NSBP1	1.61	GRHL1	1.61
CREB1	1.61	SLC6A5	1.61
CDH24	1.60	MCM9	1.61
DMC1	1.56	RGN	1.61
PHAX	1.53	CDC25B	1.61
MTSS1L	1.51	STATH	1.60

List shows the top 50 genes displaying >1.5-fold changes in expression after FLAG sorting. Predicted genes are not shown. FC, fold change.

**Table II.** Genes sharing a common feature such as homology or biochemical activity.

Gene feature	Cytokines and growth factors	Transcription factors	Homeodomain proteins	Cell differentiation markers	Protein kinases	Translocated cancer genes	Oncogenes	Tumor suppressors
Tumor suppressor	1 (1/0)	1 (0/1)	0	1	0	0	0	3 (1/2)
Oncogenes	0	3 (1/2)	0	0	0	7 (2/5)	7 (2/5)	
Translocated cancer genes	0	3 (1/2)	0	0	0	7 (2/5)		
Protein kinases	0	0	0	0	7 (1/6)			
Cell differentiation markers	0	0	0	2 (0/2)				
Homeodomain proteins	0	1 (0/1)	3 (1/2)					
Transcription factors	0	24 (8/16)						
Cytokines and growth factors	6 (2/4)							

Genes displaying >1.5-fold changes in expression after FLAG sorting were categorized according to common biological features. Predicted genes are not shown in this list. The numbers of up- and downregulated genes are presented in parentheses (up/down).
